# Octogenarian newly diagnosed multiple myeloma patients without geriatric impairments: the role of age >80 in the IMWG frailty score

**DOI:** 10.1038/s41408-021-00464-w

**Published:** 2021-04-12

**Authors:** Mattia D’Agostino, Alessandra Larocca, Massimo Offidani, Anna Marina Liberati, Gianluca Gaidano, Maria Teresa Petrucci, Daniele Derudas, Andrea Capra, Renato Zambello, Nicola Cascavilla, Paolo de Fabritiis, Vanessa Innao, Francesca Bonello, Francesca Patriarca, Giulia Benevolo, Nicola Giuliani, Gabriele Aitoro, Tommasina Guglielmelli, Francesco Di Raimondo, Paolo Corradini, Pellegrino Musto, Roman Hájek, Pieter Sonneveld, Mario Boccadoro, Sara Bringhen

**Affiliations:** 1 Myeloma Unit, Division of Hematology, University of Torino, Azienda Ospedaliero-Universitaria Città della Salute e della Scienza di Torino, Torino, Italy; 2 Clinica di Ematologia, AOU Ospedali Riuniti di Ancona, Ancona, Italy; 3 Università degli Studi di Perugia, Azienda Ospedaliera Santa Maria, Terni, Italy; 4 Division of Hematology, Department of Translational Medicine, University of Eastern Piedmont, 28100 Novara, Italy; 5 Hematology, Department of Translational and Precision Medicine, Azienda Ospedaliera Policlinico Umberto I, Sapienza University of Rome, Rome, Italy; 6 SC di Ematologia e CTMO, Ospedale Oncologico di Riferimento Regionale “A. Businco”, ARNAS “G. Brozu”, Cagliari, Italy; 7 Padova University School of Medicine, Hematology and Clinical Immunology, Padova, Italy; 8 Division of Hematology, IRCCS “Casa Sollievo della Sofferenza” Hospital, San Giovanni Rotondo, Italy; 9 Hematology, St. Eugenio Hospital ASL Roma 2, Tor Vergata University, Rome, Italy; 10 Division of Hematology, Department of Human Pathology in Adulthood and Childhood, University of Messina, 98122 Messina, Italy; 11 Clinica Ematologica e Unità di Terapie Cellulari, Azienda Sanitaria Universitaria Friuli Centrale (ASUFC), Dipartimento di Area Medica (DAME), Università di Udine, Udine, Italy; 12 SC Hematology, AO Città della Salute e della Scienza, Turin, Italy; 13 Hematology, “Azienda Ospedaliero-Universitaria” and Department of Medicine and Surgery, University of Parma, Parma, Italy; 14 Department of Medicine, Azienda Sanitaria Locale Torino 4, Cirié, Chivasso e Ivrea, Italy; 15 Ematologia, Ospedale San Luigi Gonzaga, Orbassano, Italy; 16 Division of Hematology, AOU Policlinico, University of Catania, Catania, Italy; 17 Divisione di Ematologia, Fondazione IRCCS Istituto Nazionale dei Tumori di Milano, Università degli Studi di Milano, Milano, Italy; 18 Department of Emergency and Organ Transplantation, ‘‘Aldo Moro’’ University School of Medicine, Bari, Italy; 19 Unit of Hematology and Stem Cell Transplantation, AOUC Policlinico, Bari, Italy; 20 Department of Haematooncology, University Hospital Ostrava, Ostrava, Czech Republic; 21 Faculty of Medicine, University of Ostrava, Ostrava, Czech Republic; 22 Department of Hematology, Erasmus MC Cancer Institute, Rotterdam, the Netherlands

**Keywords:** Medical research, Myeloma

Dear Editor,

In transplant-ineligible patients with newly diagnosed multiple myeloma (NDMM), impaired organ function and reduced physiological reserves may lead to a frail phenotype limiting the safe use of drugs and worsening patient outcome^1,2^.

In 2015, the International Myeloma Working Group (IMWG) has developed an index to identify frail patients based on age, Charlson Comorbidity Index (CCI), Activities of Daily Living (ADL), and Instrumental ADL (IADL)^3^. Briefly, patients are stratified according to an additive score (range 0–5) evaluating age (≤75 years = 0 points, 76–80 years = 1 point, >80 years = 2 points), CCI (≤1 = 0 points, ≥2 = 1 point), ADL (>4 = 0 points, ≤4 = 1 point), and IADL (>5 = 0 points, ≤5 = 1 point). Patients are classified as “fit” if the additive score is 0, “intermediate fit” if the additive score is 1, and “frail” if the additive score is ≥2. According to this score, patients aged >80 years are determined to be frail independently from the presence of geriatric impairments (defined as CCI ≤ 1 and/or ADL > 4 and/or IADL > 5).

Since age in itself does not necessarily define biological frailty, the aim of our analysis was to describe the outcome of NDMM patients aged >80 years without geriatric impairments.

We analyzed the original cohort that was used to define the IMWG frailty score, consisting of 869 transplant-ineligible NDMM patients enrolled in three prospective trials (EMN01, 26866138-MMY2069, and IST-CAR-506)^3–6^.

Frail patients were divided into two groups: patients who were determined to be frail by age only (Frail_by_age, i.e., patients aged >80 years with CCI ≤ 1 and ADL > 4 and IADL > 5) vs. patients who were determined to be frail for any other reason (Frail_by_other).

The median follow-up was 65 months. Fit and intermediate-fit patients were used as reference population (No_frail, *n* = 609, 70%).

Among frail patients (*n* = 260, 30%), only 70 patients were Frail_by_age (8.1%). The remaining 190 frail patients (21.9%) showed alterations in CCI (≥2 in 43% of cases), ADL (≤4 in 47% of cases), or IADL (≤5 in 58% of cases) scores and were classified as Frail_by_other. Baseline characteristics are shown in Table 1.

**Table 1:** Baseline characteristics of Frail_by_age, Frail_by_other, and No_frail patients.

Characteristics	Frail_by_age *N* = 70	Frail_by_other *N* = 190	No_frail *N* = 609	*p*-value*
**Median age** (range)	83 (81–89)	78 (67–91)	72 (50–80)	<0.001
**Age**, *n* (%)				<0.001
≤75	0	46 (24)	477 (78)	
76–80	0	96 (51)	132 (22)	
>80	70 (100)	48 (25)	0	
**CCI**, *n* (%)				<0.001
≤1	70 (100)	108 (57)	547 (90)	
≥2	0	82 (43)	62 (10)	
**ADL**, *n* (%)				<0.001
>4	70 (100)	101 (53)	579 (95)	
≤4	0	89 (47)	30 (5)	
**IADL**, *n* (%)				<0.001
>5	70 (100)	79 (42)	564 (93)	
≤5	0	111 (58)	45 (7)	
**ECOG**, *n* (%)				<0.001
≤1	60 (88)	89 (48)	507 (87)	
≥2	8 (12)	95 (52)	77 (13)	
Missing	2	6	25	
**ISS**, *n* (%)				<0.001
I	22 (31)	26 (14)	191 (31)	
II	25 (36)	88 (46)	248 (41)	
III	23 (33)	76 (40)	170 (28)	
**FISH risk**, *n* (%)				0.087
Standard	38 (64)	117 (71)	368 (76)	
High**	21 (36)	48 (29)	115 (24)	
Missing	11	25	126	

*CCI* Charlson Comorbidity Index, *ADL* Activities of Daily Living, *IADL* Instrumental Activities of Daily Living, *ECOG* Eastern Cooperative Oncology Group Performance Status, *ISS* International Staging System stage, *FISH* fluorescence in situ hybridization.

*Chi-squared test, Kruskal-Wallis test, or Fisher’s exact test as appropriate.

**Highrisk by FISH defined by the presence of t(4;14) and/or t(14;16) and/or del17p13.

As expected, Frail_by_age patients were older (median age 83) than Frail_by_other (median age 78) and No_frail patients (median age 72; *p* < 0.001).

At diagnosis, Frail_by_age patients compared to Frail_by_other patients showed a better Eastern Cooperative Oncology Group Performance Status (ECOG ≥ 2 in 12% vs. 52%, *p <* 0.001) and less advanced disease (International Staging System [ISS] stage I in 31% vs. 14%, *p* < 0.001), similarly to No_frail patients.

No differences in terms of trial distribution (EMN01 69% vs. 63%, 26866138-MMY2069 29% vs. 33%, IST-CAR-506 4% vs. 3%) and treatment received (Table S1) were found between Frail_by_age and Frail_by_other patients.

In a multivariate Cox model adjusted for ISS, cytogenetics, and treatment protocol, a worse overall survival (OS) was observed in both Frail_by_age (HR 1.51, *p* = 0.021) and Frail_by_other patients (HR 1.71, *p <* 0.001), as compared to No_frail patients (Fig. 1A). The median OS was 42.9 months in the Frail_by_age group, 41.6 months in the Frail_by_other group, and 76.5 months in the No_frail group.

**Fig. 1:**
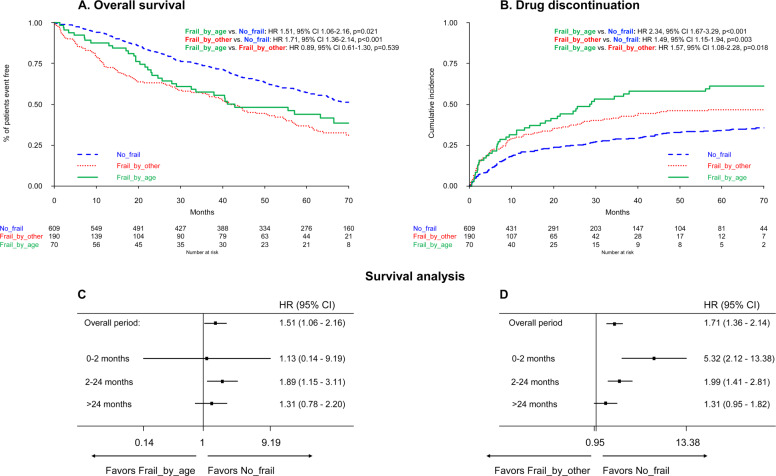
Outcomes of patients according to frailty group. Overall survival (**A**), cumulative incidence of drug discontinuation for any cause excluding progression and death (**B**), and survival analysis by time from diagnosis (**C**, **D**). OS overall survival, HR hazard ratio, CI confidence interval, *p* *p*-value.

Of note, no differences in terms of OS were found between Frail_by_age and Frail_by_other patients (HR 0.89, *p* = 0.539). Progression-free survival (PFS) and PFS2 data showed no significant differences between the two frail groups as well (Fig. S1A, B).

In the No_frail group, patients remained on study for a median time of 18.9 months, longer than patients in both the Frail_by_age (12.3 months) and Frail_by_other groups (12.4 months; Fig. S2).

Reasons for treatment discontinuation are reported in Table S2. The main reasons for discontinuation were adverse event (60%) in the Frail_by_age group, adverse event (46%) and disease progression (48%) in the Frail_by_other group, and disease progression (51%) in the No_frail group. A significantly higher risk of drug discontinuation for any cause, excluding progression and death, was reported in the Frail_by_age group (HR 2.34, *p* < 0.001) and Frail_by_other group (HR 1.49, *p* = 0.003), as compared to the No_frail group (Fig. 1B). Frail_by_age patients had a higher risk of drug discontinuation compared to Frail_by_other patients as well (HR 1.57, *p* = 0.018). Nevertheless, the cumulative incidence of grade ≥3 non-hematologic and hematologic toxicities was not significantly different between Frail_by_age and Frail_by_other patients (Fig. S3A, B).

At the current follow-up, a second therapy was started in 61% vs. 66% vs. 72% of patients in Frail_by_age vs. Frail_by_other vs. No_frail groups, respectively. Among second therapies, low-dose conventional chemotherapy without novel agents was used in 37% vs. 24% vs. 7% of Frail_by_age vs. Frail_by_other vs. No_frail patients (*p* < 0.001), suggesting that patients aged >80 years were more likely to receive a suboptimal therapy after the first line.

We next analyzed OS by dividing the time from diagnosis into three time frames to account for early deaths (0–2 months)^7^, deaths within 2 years from diagnosis (2–24 months), and late deaths (>24 months; Fig. 1C, D).

No significant differences in terms of early deaths were found between the Frail_by_age (HR 1.13, *p* = 0.909) and the No_frail groups, whereas a higher risk of early death was observed in the Frail_by_other group (HR 5.32, *p* < 0.001). Within the first 2 months, 21/869 patients died overall (2%), while this percentage was significantly higher in the Frail_by_other group (13/190, 7%). The main cause of death in this time frame was death due to toxicity (62%).

Between 2 and 24 months from diagnosis, both Frail_by_age (HR 1.89, *p* = 0.012) and Frail_by_other (HR 1.99, *p* < 0.001) patients showed a significantly higher risk of death, as compared to No_frail patients. In this time frame, the main cause of death was progressive disease (65%), followed by toxicity (24%).

No differences in terms of late deaths were observed among the three groups. The main cause of late death was progressive disease (59%), followed by toxicity (22%).

To exclude an OS bias due to the older age of Frail_by_age patients, we explored the impact of geriatric impairments on patients aged ≤80 years and >80 years (Fig. S4A, B). The presence of geriatric impairments significantly predicted a lower OS in the population aged ≤80 years (HR 1.46, *p* < 0.001), but not in the population aged >80 years (HR 1.09, *p* = 0.739), thus supporting the hypothesis that octogenarian NDMM patients are frail independently from the presence of geriatric impairments.

To summarize our findings, octogenarian patients without geriatric impairments usually present with a low disease burden and a good performance status. However, the high rate of drug discontinuations and the difficulty to deliver effective treatments after the first line of treatment may lead to the observed poor survival.

To date, this patient population is rare, accounting for <10% of NDMM patients in clinical trials. Nevertheless, the life expectancy and health conditions of the general population are improving^8^. Thus, in the near future, physicians are expected to face a growing percentage of octogenarian NDMM patients without geriatric impairments^9^.

New treatments (e.g., naked monoclonal antibodies) that can be safely delivered continuously for a long period of time may be better tolerated and have a lower discontinuation risk, potentially improving the outcome of this patient population. Indeed, dedicated trials selectively enrolling intermediate-fit and/or frail patients are beginning to emerge^10,11^. In a randomized phase III trial in intermediate-fit patients, 9 cycles of lenalidomide-dexamethasone induction followed by low-dose lenalidomide maintenance without steroids produced similar outcomes compared to continuous lenalidomide-dexamethasone (median PFS 20.2 vs. 18.3 months), thus showing that, in this patient subgroup, therapy could be de-intensified after induction without affecting patient outcome. Another trial enrolling both intermediate-fit and frail patients explored daratumumab-ixazomib-dexamethasone induction followed by daratumumab-ixazomib maintenance. A total of 70% of intermediate-fit and 61% of frail patients completed induction treatment (9 months) and PFS rates were 78% and 61%, respectively. The early death rate (≤3 months after study entry) was higher in frail patients than in intermediate-fit patients (12% vs. 0%).

Interestingly, in our work, we observed an excess of early toxic deaths (<2 months from diagnosis) in patients who were frail due to geriatric impairments. This observation may support the exploration of dose-escalation strategies in the first months after diagnosis in frail NDMM patients presenting with geriatric impairments.

In conclusion, in this work, we showed that NDMM patients who were frail by age >80 years but who did not present with any geriatric impairments had a similar OS compared to patients who were determined to be frail for any other reason. These data further support that NDMM patients aged >80 years should be classified as frail regardless of the presence/absence of any comorbidities and ADL/IADL limitations.

## Supplementary information

Supplementary Appendix

aj-checklist - Frail by age letter
