# Supplementary material for: Octogenarian newly diagnosed multiple myeloma patients without geriatric impairments: the role of age >80 in the IMWG frailty score
Source: Blood Cancer J. 2021 Apr 12;11(4):73. doi: 10.1038/s41408-021-00464-w (PMC8041817; doi:10.1038/s41408-021-00464-w)
Supplement: Supplementary file 1 — Supplementary Appendix [file 41408_2021_464_MOESM1_ESM.pdf]

Octogenarian Newly Diagnosed Multiple Myeloma Patients Without Geriatric Impairments: The Role of Age >80 in the IMWG Frailty Score

SUPPLEMENTARY APPENDIX

- **Table S1**. Treatment received by Frail_by_age and Frail_by_other patients
- **Table S2**. Treatment discontinuation details
- **Figure S1**. PFS (A) and PFS2 (B) of Frail_by_age vs. Frail_by_other patients. No_frail patients are shown as reference.
- **Figure S2**. Time to protocol discontinuation (end of treatment) of Frail_by_age vs. Frail_by_other patients. No_frail patients are shown as reference.
- **Figure S3**. Cumulative incidence of grade ≥3 non-hematologic (A) and hematologic (B) adverse events
- **Figure S4**. OS of patients aged ≤80 years (A) and >80 years (B) according to the presence or absence of geriatric impairments

Table S1. Treatment received by Frail_by_age and Frail_by_other patients

Treatment	Frail_by_age N=70 (%)	Frail_by_other N=190 (%)
**CCD**	2 (3)	8 (4)
**CPR**	12 (17)	42 (22)
**MPR**	16 (23)	33 (17)
**Rd**	20 (29)	45 (24)
**VMP**	6 (9)	19 (10)
**VCP**	3 (4)	16 (8)
**VP**	11 (16)	27(14)

Abbreviations. CCD, carfilzomib-cyclophosphamide-dexamethasone; CPR, cyclophosphamide-prednisone-lenalidomide; MPR, melphalan-prednisone-lenalidomide; Rd, lenalidomide-low-dose dexamethasone; VMP, bortezomib-melphalan-prednisone; VCP, bortezomib-cyclophosphamide-prednisone; VP, bortezomib-low-dose prednisone.

Table S2. Treatment discontinuation details

Discontinuation	Frail_by_age N=70 (%)	Frail_by_other N=190 (%)	No_frail N=609 (%)
**Adverse event**	42 (60)	88 (46)	215 (35)
Hematologic	5 (7)	8 (4)	27 (4)
Dermatologic	4 (6)	2 (1)	22 (4)
Infections	3 (4)	12 (6)	12 (2)
Cardiac	3 (4)	8 (4)	13 (2)
Neurologic	2 (3)	4 (2)	10 (2)
**Progressive disease**	23 (33)	91 (48)	313 (51)
**Other**	5 (7)	11 (6)	81 (13)

Figure S1. PFS (A) and PFS2 (B) of Frail_by_age vs. Frail_by_other patients. No_frail patients are shown as reference.

A. PFS

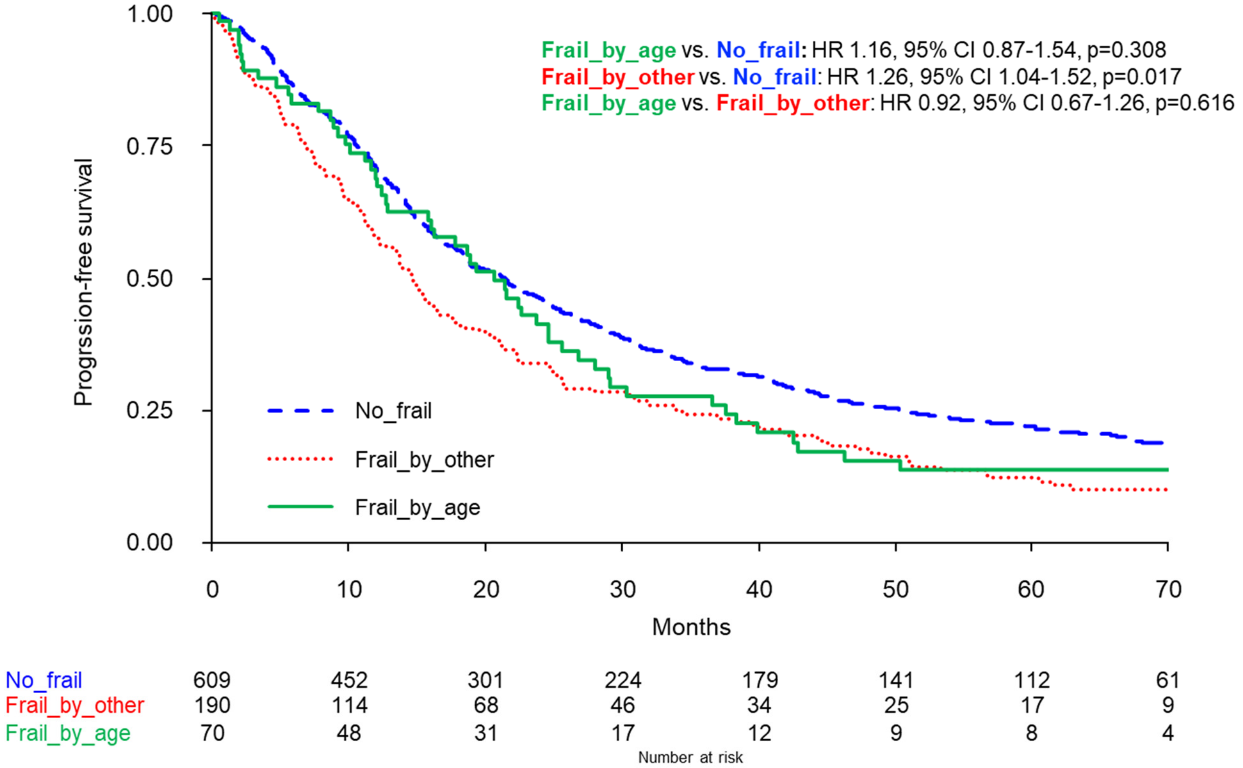

B. PFS2

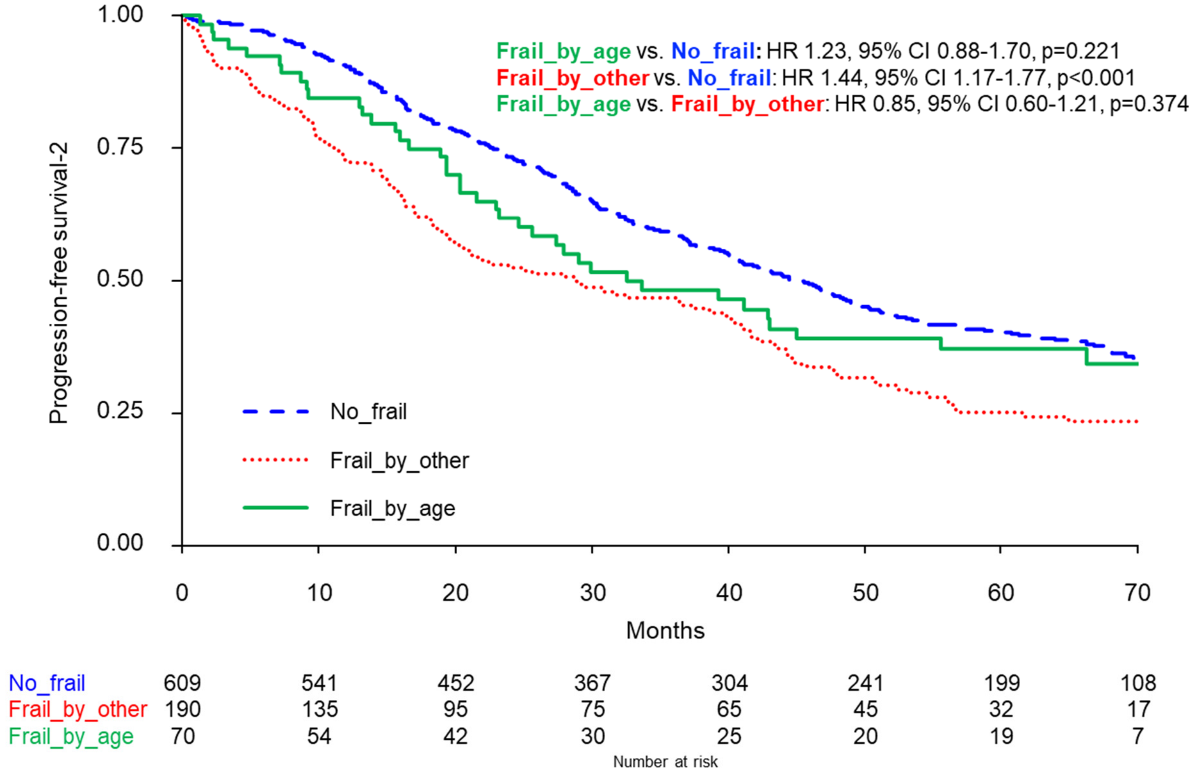

<u>Abbreviations</u>. PFS, progression-free survival; PFS2, progression-free survival-2; HR, hazard ratio; CI, confidence interval; p, p-value.

Figure S2. Time to protocol discontinuation (end of treatment) of Frail_by_age vs. Frail_by_other patients. No_frail patients are shown as reference.

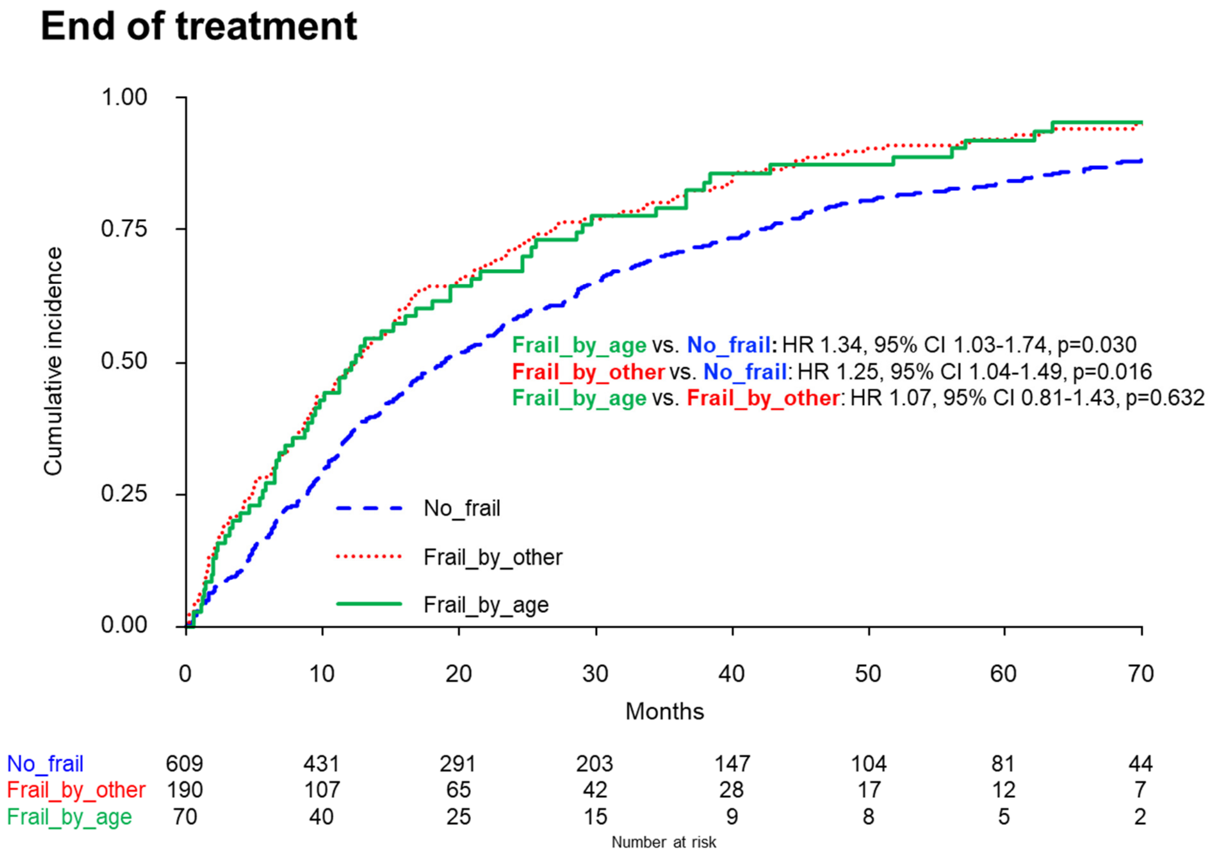

Abbreviations. HR, hazard ratio; CI, confidence interval; p, p-value.

Figure S3. Cumulative incidence of grade ≥3 non-hematologic (A) and hematologic (B) adverse events

A. Grade ≥3 non-hematologic adverse events

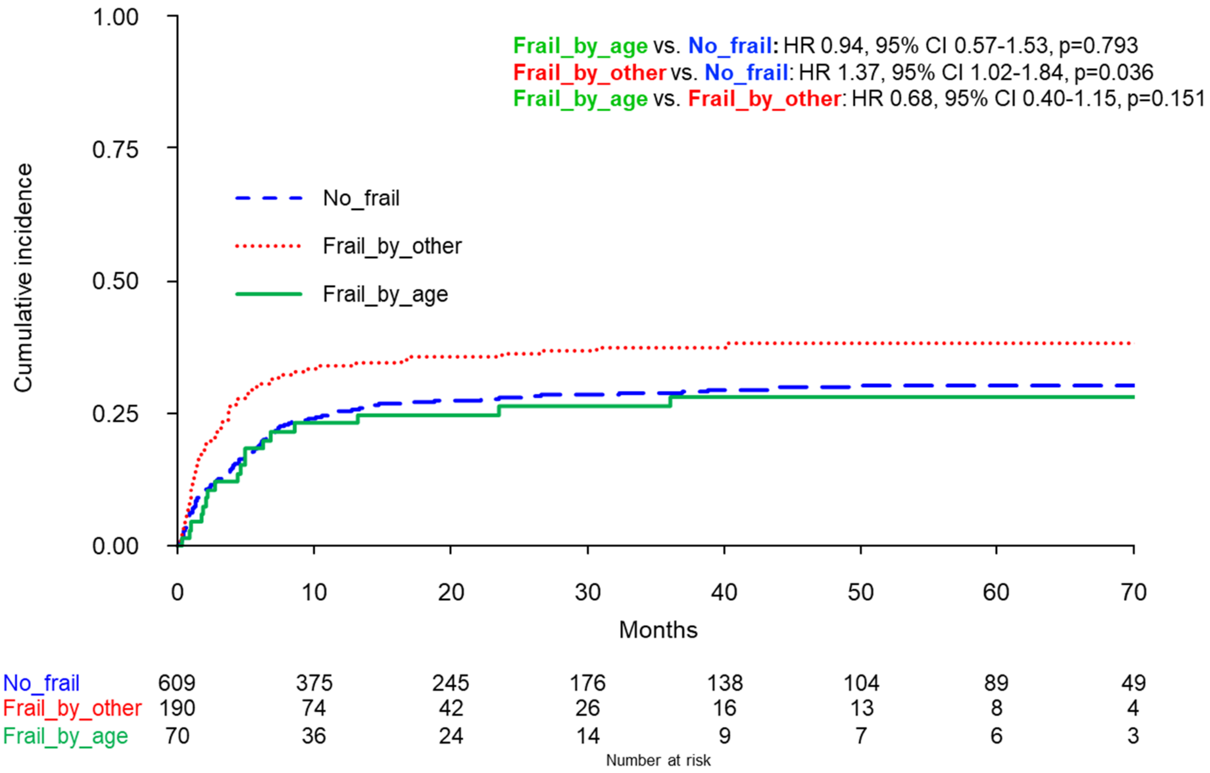

B. Grade ≥3 hematologic adverse events

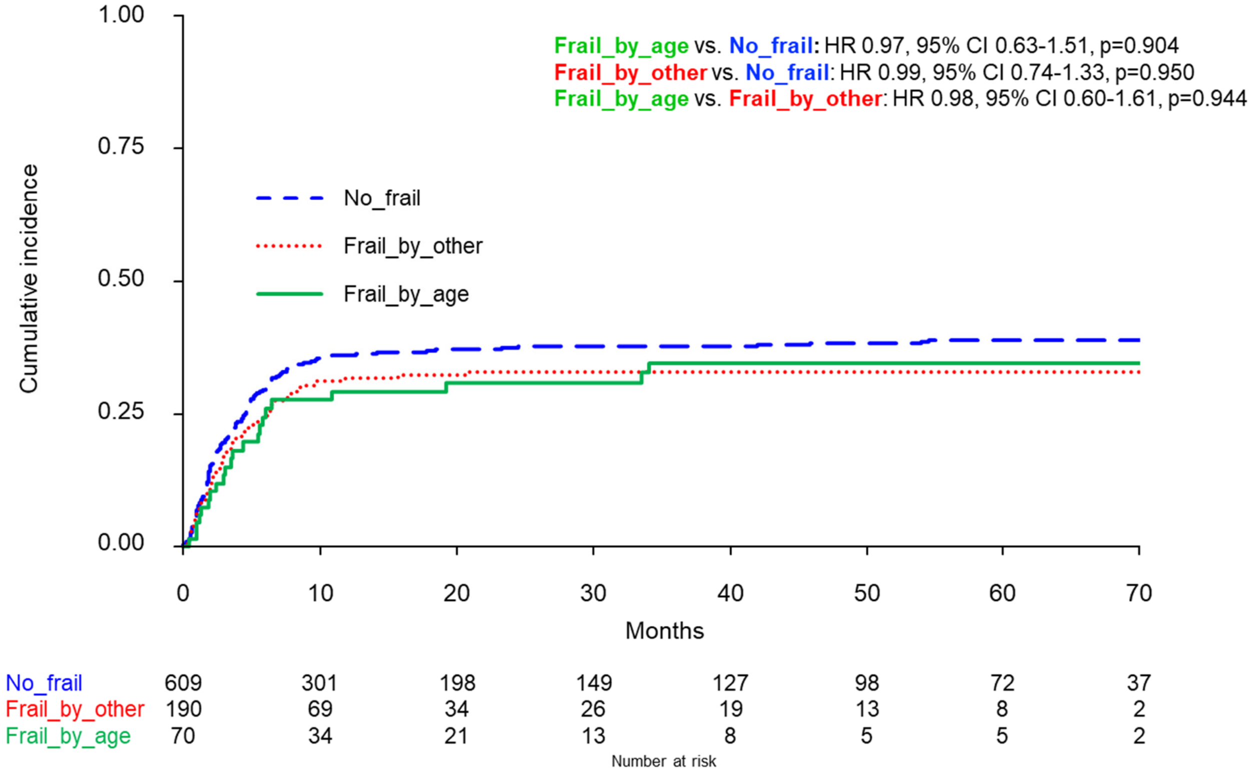

Abbreviations. HR, hazard ratio; CI, confidence interval; p, p-value.

Figure S4. OS of patients aged ≤80 years (A) and >80 years (B) according to the presence or absence of geriatric impairments

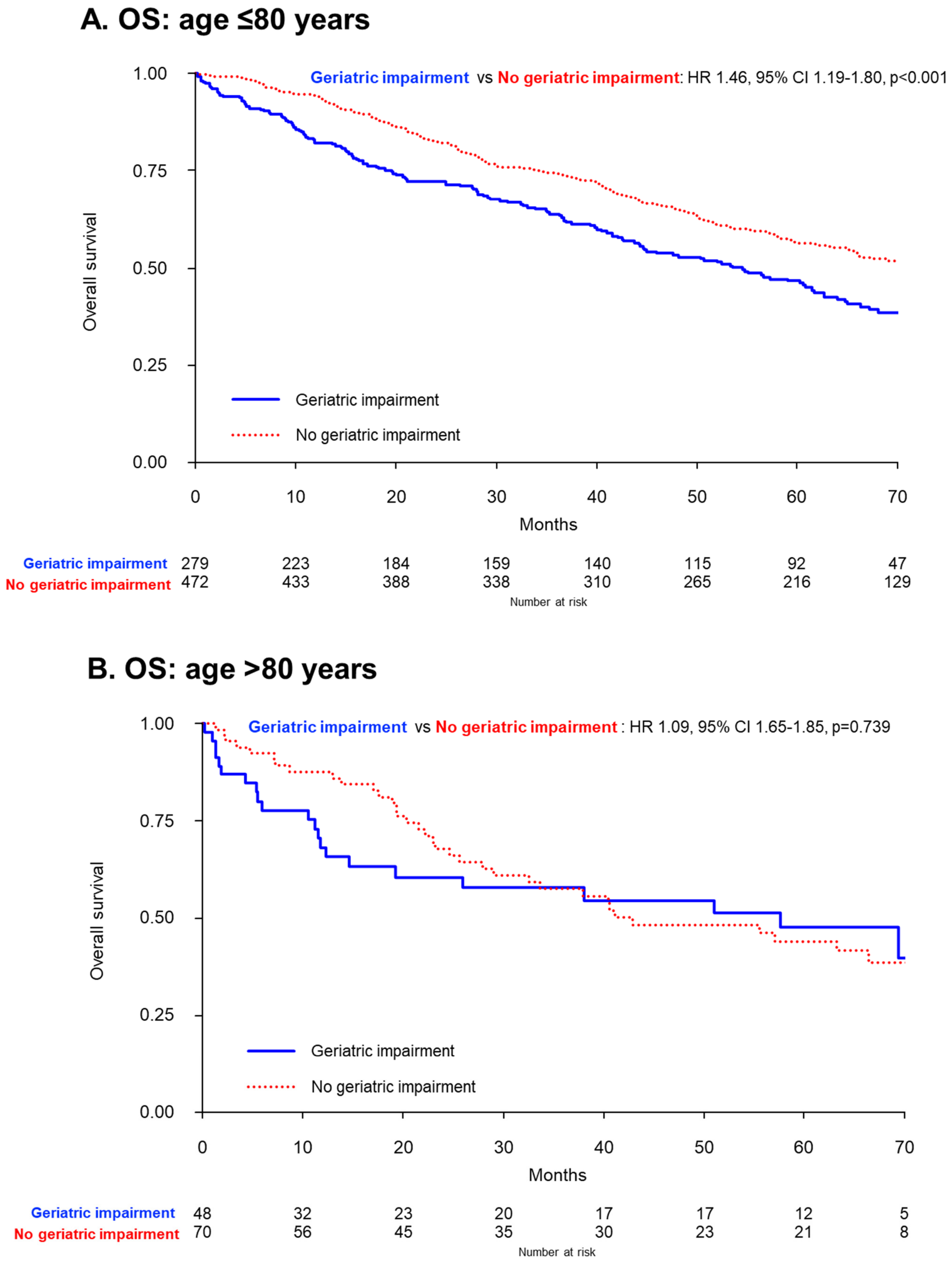

Abbreviations. OS, overall survival; HR, hazard ratio; CI, confidence interval; p, p-value.
